# Efficacy of Felpreva®, a new spot-on formulation containing tigolaner, emodepside and praziquantel, applied as a single application to cats artificially infested with ear mites (*Otodectes cynotis*)

**DOI:** 10.1016/j.crpvbd.2023.100131

**Published:** 2023-07-03

**Authors:** Katrin Blazejak, Alta Viljoen, Reinier Zwiegers, Roland Klopper, Hannah Ringeisen, Gabriele Petry, David R. Young, Douglas Shane, Jennifer Spruill, Ronald K. Tessman, Terry Settje, Tanja N. Knoppe, Norbert Mencke

**Affiliations:** a Vetoquinol S.A., 37 Rue de la Victoire, 75009 Paris, France; b Clinvet International (Pty) Ltd, PO Box 11186, 9321 Universitas, South Africa; c Clindata International (Pty) Ltd, PO Box 11186, 9321 Universitas, South Africa; d Elanco Animal Health Company, Alfred Nobel Str. 50, 40789 Monheim, Germany; e Young Veterinary Research Services, 3000 Spengler Way, Turlock, CA 95380, USA; f Elanco Animal Health, 2500 Innovation Way, Greenfield, IN 46140, USA; g Vet Advice, Dornstücken 25, 22607 Hamburg, Germany

**Keywords:** Tigolaner, Felpreva®, Ear mite, *Otodectes cynotis*, Cat, Otoacariosis

## Abstract

The efficacy of Felpreva® (Vetoquinol), a new spot-on application containing the novel acaricide and insecticide tigolaner in combination with emodepside and praziquantel, was evaluated in cats artificially infested with ear mites (*Otodectes cynotis*). A total of three pivotal dose confirmation studies were conducted, two of them designed as non-interference studies. Cats were artificially infested with *O. cynotis* mites and randomly allocated into groups of 8 cats based on pre-treatment mite counts. Cats were treated once on Day 0, either with Felpreva® (14.5 ​mg/kg tigolaner, 3 ​mg/kg emodepside and 12 ​mg/kg praziquantel) or with placebo. Studies with a non-interference design included two additional groups of cats, treated with Profender® spot-on solution (Vetoquinol) (3 ​mg/kg emodepside and 12 ​mg/kg praziquantel) and tigolaner as a mono product (14.5 ​mg/kg tigolaner). Efficacy was evaluated on Day 28/Day 30 based on total live mite counts after ear flushing. Efficacy was claimed when: (i) at least six control cats per group were adequately infested with mites; (ii) calculated efficacy was ≥ 90% based on geometric mean mite counts; and (iii) the difference in mite counts between Felpreva®-treated cats and control cats was statistically significant (*P* ​≤ ​0.05). In two of the three studies, Felpreva®-treated cats were mite-free (100% efficacy) on Day 28/Day 30 and almost full efficacy (99.6%) was seen in the third study. The difference in mite counts between Felpreva®-treated cats and control cats was significant (*P* ​< ​0.0001) in all three studies. All control cats were adequately infested in all three studies. The efficacy of Felpreva® against ear mite (*Otodectes cynotis*) infection in cats was confirmed.

## Highlights

• Topical treatment with Felpreva® was highly effective in clearing *O. cynotis* infestations in cats 4 weeks after treatment.
• Treatment with Felpreva® was well tolerated in cats.
• Tigolaner is a new acaricide licensed for cats with a fast onset of efficacy against ectoparasites (fleas, ticks and mites).

## Graphical abstract

**Figure:**
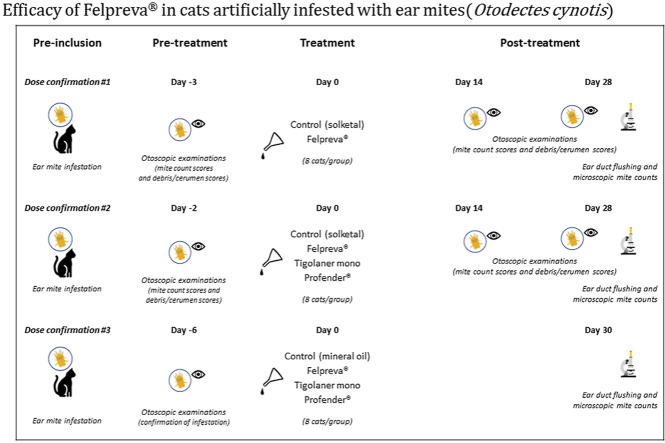

## 1 Introduction

Otoacariosis caused by the ear mite *Otodectes cynotis* (family Psoroptidae) is frequently found in cats and dogs but does also occur in wild carnivores such as wild cats, foxes, ferrets (Lohse et al., 2002). Ear mites live in the horizontal and vertical ear canal and are occasionally found outside the ear producing pruritic papular skin lesions, often on head, feet and tail tip (Bowman et al., 2002; Curtis, 2004). Off-host, ear mites seem to survive only for a couple of days. Survival times of 12 days were found under natural conditions at temperatures of 12.3–14.2 ​°C which were linearly declining with increasing temperatures (Otranto et al., 2004). *Otodectes cynotis* are non-borrowing mites that feed on epidermal debris and tissue fluids. The life-cycle is approximately 18–28 days (Bowman et al., 2002; Curtis, 2004) and the entire development (egg, larva, nymph and adult) takes place in the ear canal of the host (Muller and Kirk, 2012; Yang and Huang, 2016). Clinical signs in infested animals typically include dark-brown, ceruminous exudate and erythema inside the ear canal in combination with varying degree of pruritus (Yang and Huang, 2016). In cats however, the disease is highly variable, and severity of signs does not necessarily correlate with the number of mites present (Bowman et al., 2002). Affected cats can present anything from apparently healthy (Sotiraki et al., 2001) to severe otitis externa (Yang and Huang, 2016). Ear mite infestations are the most common cause for feline otitis externa, accounting for approximately 50–85% of all clinical cases (Wall and Shearer, 2001; Jacobson, 2002; Brame and Cain, 2021). The disease is highly contagious and affects all types of cats (Noli, 2020). Transmission of *O. cynotis* occurs by direct contact, often from infected mothers to their kittens. Factors like geographical region, age, multi-pet households or frequent access to other cats or hosts can be risk factors (Fanelli et al., 2020). The prevalence of ear mites in cats is variable, though epidemiological data are scarce. In a European multicenter survey, *O. cynotis* was diagnosed in 17.4% of client-owned cats and cats with regular outdoor access had a higher risk for ear mite infestations than cats with only infrequent outdoor access (Beugnet et al., 2014). In a survey from Greece, 14% of kittens and young cats presented to veterinarians were infested with *O. cynotis* (Lefkaditis et al., 2009), whereas in another study, ear mites were found in 25.5% of adult cats (Sotiraki et al., 2001**)**. In stray cats from Portugal, the prevalence was 2.2% (Duarte et al., 2010), whereas the prevalence in shelter cats from Spain was 30% (Fanelli et al., 2020). In a cohort of client-owned, shelter and colony cats from Italy, ear mites were found in 9.8% of the cats (Genchi et al., 2021). When ear mites are diagnosed in an animal, it is generally assumed that all contact animals are infested and treatment recommendations include to treat all susceptible pets in a multi-animal household (CAPC, 2019; ESCCAP, 2022).

Felpreva® is a new broad-spectrum spot-on formulation, which was recently registered for cats in Europe, containing tigolaner, emodepside, and praziquantel at the minimum recommended dose of 14.4 mg/kg, 3 ​mg/kg, and 12 ​mg/kg body weight, respectively (EMA, 2021). The product has been demonstrated to have high anthelmintic activity in cats (Cvejić et al., 2022a; Traversa et al., 2022) and high efficacy against common tick and flea species in Europe (Cvejić et al., 2022b), in combination with a fast onset of flea kill (Mencke et al., 2023). The purpose of this article is to present the miticidal activity of the product. Three laboratory studies in cats experimentally infested with *O. cynotis* were conducted. The objective of these studies was to test whether a single topical application of Felpreva® spot-on is effective in eliminating ear mite infestations in cats until four weeks after treatment.

## 2 Materials and methods

A total of three pivotal dose confirmation studies were conducted, two of them (study #2 and #3) designed as non-interference studies. Two study sites were involved, one was located in the Republic of South Africa (study #1 and #2) and one was located in the USA (study #3). All three studies were in compliance with VICH GL 9 Principles of Good Clinical Practice (EMA, 2000) and internal Standard Operating Procedures (SOPs). The studies were designed following the recommendations of guidelines “Demonstration of efficacy of ectoparasiticides” (EMA, 1994) and “Testing and evaluation of the efficacy of antiparasitic substances for the treatment and prevention of tick and flea infestation in dogs and cats” (EMA, 2016). All studies were part of a development programme for the regulatory approval of Felpreva®.

### 2.1 Animals and study design

The studies were randomised, blinded, negative controlled studies, using a parallel group design. Study animals were purpose-bred Domestic Shorthair cats (*Felis catus*) of both sexes, between 8 and 172 months of age and with body weights ranging between 2.2 and 5.9 ​kg. The cats were housed individually after Day 0, according to accepted local animal welfare regulations (South African National Standard SANS 10386 for study #1 and #2; US Department of Agriculture USDA Animal Welfare Regulations for study #3), and Ethics Committee approvals, where applicable. Cats were fed standard commercial diets appropriate for their age and nutritional needs. Water was supplied *ad libitum*. Food and water were expected to be free of any contaminants that could interfere with the study.

All cats were acclimatised for at least 7 days and were clinically healthy at study start. None of the cats had been treated with any topical or systemic acaricide/insecticide before study inclusion that could have interfered with the study objectives. Approximately 1 month before study inclusion, the cats were artificially infested with live *O. cynotis* mites harvested from donor cats. Success of the artificial infestation was verified by otoscopic examination between Day -6 and Day -2. Live mites were counted in both ears of each cat using a scoring system from 0 (no live mites) to 3 (> 10 live mites) (pre-treatment mite counts).

Cats were blocked within sex on individual pre-treatment mite counts and then randomly allocated to treatment groups. Each study group included 8 cats (males and females) per group. All personnel performing mite count evaluations and involved in general, in clinical and in local tolerance observation procedures were blinded to treatment allocations.

Body weights were determined pre-treatment (between Day -3 and Day -1) for dose calculation purposes and reassessed at study end on Day 28 (study #1 and #2) and Day 30 (study #3). Physical exams were performed pre-treatment (Day -9/Day -2) and at study completion (Day 28/Day 30). General health observations were made daily from Day -7 until study end. Clinical examinations post-treatment were conducted at 1 ​h (± 15 ​min), 2 ​h (± 15 ​min), 4 ​h (± 30 ​min), 6 ​h (± 1 ​h) and 8 ​h (± 30 ​min), and again on Days 1, 2 and 7. Treatment site evaluations were made shortly before treatment application and again at 1 ​h (± 15 ​min), 2 ​h (± 15 ​min), 4 ​h (± 30 ​min), 6 ​h (± 30 ​min), and 8 ​h (± 30 ​min) post-treatment, and again on Days 1, 2, 7, 14, 21, and 28.

### 2.2 Treatment administrations

Dose regimens of all three studies are displayed in Table 1. In study #1, one group of cats was treated with Felpreva® (at the minimum recommended dose of 14.5 ​mg tigolaner, 3 ​mg emodepside and 12 ​mg praziquantel per kg body weight) and the other group with placebo (solketal, syn. isopropylidineglycerol, a glycerol derivative). In study #2 and #3, groups of cats were assigned to one of the following treatments: (i) Felpreva® (Vetoquinol S.A., France) at the minimum recommended dose; (ii) tigolaner mono spot-on at a dose of 14.5 ​mg per kg body weight; (iii) Profender® (Vetoquinol S.A., France) at the minimum recommended dose of 3 ​mg emodepside and 12 ​mg praziquantel per kg body weight; or (iv) placebo (solketal or mineral oil). The dose volumes were the same for all products including placebo (0.148 ​ml per kg bodyweight).

**Table 1:** Design of Felpreva® dose confirmation studies in cats artificially infested with ear mites *Otodectes cynotis*.

Study	Product	Actives and minimum dose rates per kg BW	Dose volume (ml/kg BW)	Ear mite counts
#1	Solketal	na	0.148	Days -3, 14 and 28
	Felpreva®	14.5 ​mg/kg tigolaner ​+ ​3 ​mg/kg emodepside ​+ ​12 ​mg/kg praziquantel	0.148	
#2	Solketal	na		Days -2, 14 and 28
	Felpreva®	14.5 ​mg/kg tigolaner ​+ ​3 ​mg/kg emodepside ​+ ​12 ​mg/kg praziquantel	0.148	
	Tigolaner mono	14.5 ​mg/kg tigolaner	0.148	
	Profender	3 ​mg/kg emodepside ​+ ​12 ​mg/kg praziquantel	0.148	
#3	Mineral oil	na	0.148	Days -6 and 30
	Felpreva®	14.5 ​mg/kg tigolaner ​+ ​3 ​mg/kg emodepside ​+ ​12 ​mg/kg praziquantel	0.148	
	Tigolaner mono	14.5 ​mg/kg tigolaner	0.148	
	Profender	3 ​mg/kg emodepside ​+ ​12 ​mg/kg praziquantel	0.148	

*Abbreviations*: BW, body weight; na, not applicable.

Application volumes (calculated as pre-treatment body weight ​× ​dose volume per kg body weight) were rounded up to two decimal places. All products were administered once on Day 0, applied as spot-on formulations directly to the skin at the base of skull of each cat.

### 2.3 Ear mite infestations, ear mite counts and debris/cerumen score

*Otodectes cynotis* mites used for the experimental infestation procedures were local isolates, one originating from naturally infested cats in South Africa (study #1 and #2) and one originating from naturally infested cats in the USA (study #3). Before study start, mites were harvested from donor cats by lavage with saline solution or by use of cotton swabs. The mites were then deposited into both ear canals of each study cat, either on a tuft of hair or directly. Depending on study site and the experimental model that was used, the infestation dose was at least 80–100 live mites/ear (study #1 and #2) or at least 10 live mites/ear (study #3). Study cats were sedated during the procedure to prevent head shaking and removal of the infestation material from the ears during the first hours after infestation.

Regular qualitative otoscopic examinations were performed pre-treatment on both ears of all cats to verify the infestation success. Cats were eligible for study inclusion when the presence of live ear mites was confirmed; in both ears with at least one ear presenting a minimum of 11 live mites on Day -3 (study #1 and #2) or at least one ear with a minimum of 5 live ear mites on Day -6 (study #3).

Post-treatment mite counts after ear duct flushing were used for the primary efficacy evaluation and performed at study completion on Day 28 (study #1 and #2) or Day 30 (study #3). For the procedure, cats were sedated (medetomidine hydrochloride, Dormitor®, Zoetis, 0.08 ​ml/kg and ketamine, Anaket-V, Bayer Animal Health, 0.05 ​ml/kg in study #1 and #2; xylazine and ketamine in study #3) and both ear ducts were filled with Docusol® (5% aqueous solution of docusate sodium, Kyron Laboratories). The ears were massaged lightly to loosen cerumen deposits. Dissolved solution was collected over a 38 ​μm sieve and the ear canal was flushed repeatedly with warm saline solution until it was considered clean. All collected material from one ear was transferred into a labelled container and microscopically examined. Live mites (larvae, nymphs and adult mites) of both ears were counted, summed up for each animal and recorded as the animal’s total ear mite count (quantitative assessment).

In study #1 and #2, additional qualitative assessments were performed on Day 14 and Day 28 which were used for secondary efficacy evaluations. Otoscopic mite counts were made for both ears of each cat using a mite count scoring system of 0 (no live mites), 1 (1–4 live mites), 2 (5–10 live mites), and 3 (> 10 live mites). In addition to mite counts, clinical signs of ear mite infestation were assessed in both ears, using a debris and cerumen score of 0 (no debris/cerumen), 1 (slight debris/cerumen), 2 (moderate debris/cerumen), and 3 (severe debris/cerumen).

### 2.4 Statistical analysis

Adequacy of infestation was achieved in the placebo (negative control) groups when at least 6 cats were infested with ≥ 11 ear mites (study #1 and #2) or ​≥ ​10 ear mites (study #3) as a sum of both ears.

The primary efficacy criterion was the efficacy against ear mites. The total number of live ear mite counts on Day 28/Day 30 after ear flushing were used to calculate geometric means (count ​+ ​1 data with 1 subsequently subtracted from result). Efficacy (%) was calculated using the Abbott formula: 100 ​× ​(C – T)/C, where C is the geometric mean of live mite counts of cats in the negative control group and T is the geometric mean of live ear mite counts of cats in the treated groups. Group comparisons were made using a one-way analysis of variance (ANOVA) in SAS 9.3 and higher (SAS Institute Inc., Cary, NC, USA), including treatment as a fixed effect. All hypotheses were tested at a two-sided 0.05 level of significance. Efficacy was claimed when efficacy ≥ 90% was calculated and a statistically significant difference (*P* ​≤ ​0.05) between the treatment group and control group was demonstrated. The experimental unit was the individual cat.

Secondary efficacy criteria were evaluated in study #1 and #2, which included otoscopic live mite count reductions and otoscopic improvement of debris/cerumen scores on Day 14 and Day 28. The effect of treatment was assessed for both parameters, by comparing scores (mite count score and debris/cerumen score) on Day 14 and Day 28 with scores at baseline (Day -2/Day -3). Calculations were made using the ear with the higher score of each cat. Differences were assessed using a two-sided, non-parametric test (Cochran-Mantel-Haenszel test) with the 0.05 level of significance.

## 3 Results

### 3.1 Efficacy

In all three studies, control cats were adequately infested with ear mites at study end (Day 28/Day 30). The geometric mean mite counts in the negative controls were 111.9 (range 21–881) in study #1, 103.0 (range 15–761) in study #2, and 63.2 (range 11–190) in study #3 (Table 2).

**Table 2:** Geometric mean mite counts and calculated percent efficacy against ear mites (*Otodectes cynotis*) for treated groups compared to negative controls four weeks after treatment (Day 28/30, 8 cats per group).

Study	Product	Study day	Efficacy (%)	Mean mite counts (geometric mean)	Range	*P*-value
#1	Control group	Day 28	na	111.9	21–881	na
	Felpreva®		100	0	0	<0.0001
#2	Control group	Day 28	na	103.0	15–761	na
	Felpreva®		99.6	0.5	0–4	<0.0001
	Tigolaner mono		99.9	0.1	0–1	<0.0001
	Profender®		31.6	70.4	9–337	0.4621
#3	Control group	Day 30	na	63.2	11–190	na
	Felpreva®		100	0	0	<0.0001
	Tigolaner mono		100	0	0	<0.0001
	Profender®		36.0	40.4	10–550	nd

*Abbreviations*: nd, not determined; na, not applicable.

No ear mites were recovered from cats treated with Felpreva® after ear duct flushing at study end in all three studies, except for two cats in study #2 which had 3 and 4 live mites in one ear on Day 28. Efficacy rates were 100% in study #1 and #3; and 99.6% in study #2. The difference of mite counts between Felpreva®-treated cats and control cats was statistically significant (*P* ​< ​0.0001) in all three studies.

Similar results were observed when cats were treated with tigolaner. Efficacy for tigolaner mono spot-on was 99.9% in study #2 (Day 28) and 100% in study #3 (Day 30). A single mite was found in one cat in study #2. All other tigolaner-treated cats were free of ear mites. Mite count reductions in tigolaner-treated cats were statistically significant (*P* ​< ​0.0001).

Efficacy against mites was low when cats were treated with Profender®. Efficacy rates were 31.6% in study #2 and 36.0% in study #3. Mite counts in Profender®-treated cats were not statistically different from mite counts in control cats (study #2).

**Fig. 1:**
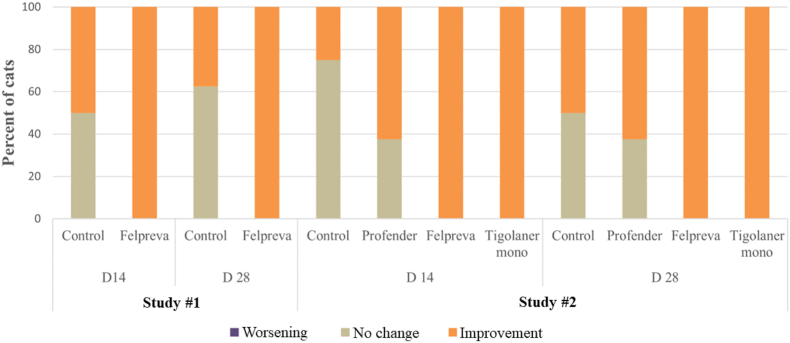
Changes of post-treatment *Otodectes cynotis* visible live mite count scores in relation to pre-treatment assessed by otoscopic examination (effect of treatment based on worst case score between both ears on Day 14 and Day 28 *versus* pre-treatment (Day -2/Day -3) in comparison to placebo, Cochran-Mantel-Haenszel test, level of significance ​= ​0.05).

Comparisons of pre- and post-treatment mite count scores by otoscopic examinations in study #1 and #2 showed that mite count scores of all (100%) Felpreva®- and all (100%) tigolaner-treated cats had improved on Day 14, as well as on Day 28, which was significantly different from control cats on both days and for both studies. The percentage of improved control cats ranged from 37.5% (Day 28) to 50% (Day 14) in study #1 and from 25% (Day 14) to 50% (Day 28) in study #2. The percentage of cats with improved mite count scores after treatment with Profender® (62.5% on both days) was not significantly different compared to control cats (Fig. 1).

**Fig. 2:**
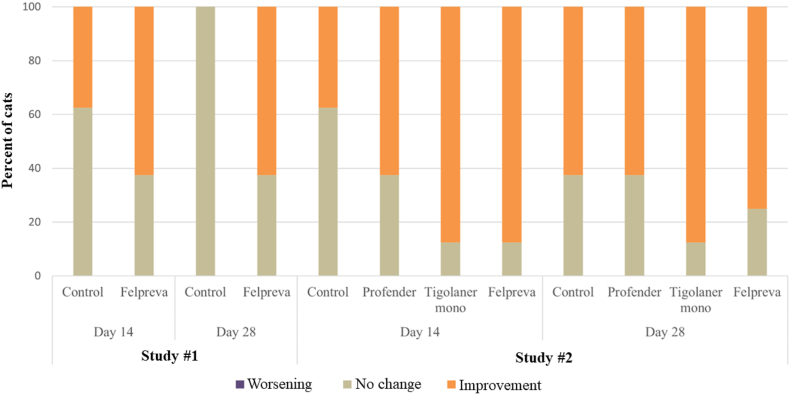
Changes of post-treatment debris/cerumen scores in relation to pre-treatment assessed by otoscopic examination (effect of treatment based on worst case score between both ears on Day 14 and Day 28 *versus* pre-treatment (Day -2/Day -3) in comparison to placebo, Cochran-Mantel-Haenszel test, level of significance ​= ​0.05).

Debris and cerumen scores were also improved after treatment with Felpreva®, though less frequently compared to mite count scores. In study #1, 62.5% of Felpreva®-treated cats had improved debris and cerumen scores on both study days whereas in study #2, improved scores were observed in 75% (Day 28) to 87.5% (Day 14) of the cats. When cats were treated with tigolaner alone (study #2), 87.5% of the cats showed improved scores (both days). In comparison, the percentage of improved control cats ranged from 0% (Day 28) to 37.5% (Day 14) in study #1 and from 37.5% (Day 14) to 62.5% (Day 28) in study #2. A significant difference in debris and cerumen scores between Felpreva®-treated cats and control cats was only demonstrated for Day 28 in study #1 and for Day 14 in study #2 (Fig. 2).

### 3.2 Safety observations

In study #2, six cases of mild erythema at the application site were observed 2 ​h post-treatment. Cases involved three control cats, two cats treated with Felpreva® and one tigolaner-treated cat. While the location of the erythema indicated a possible product relation, involvement of tigolaner was considered unlikely as the erythema was found in both, tigolaner-treated cats as well as control cats (treated with solketal). The erythema was transient and resolved 4 ​h post-treatment in all six cats. No other adverse events in relation to treatment were found in this study and no adverse events were reported in study #1 and #3. Overall, the topical application of Felpreva® was well tolerated in cats.

## 4 Discussion

Results of the three studies demonstrated that treatment with Felpreva® was highly effective in clearing ear mite infestations in cats. A single topical application at the minimum recommended dose to cats artificially infested with *O. cynotis* provided complete parasitological cure (100% efficacy) in two of three studies and almost full efficacy (99.6%) in the third study, when assessed four weeks after treatment. Ear mites were observed in two cats in the third study, but only low number of mites (3 and 4 live mites, respectively) were recovered after ear duct flushing on Day 28.

In otoscopic examinations on Day 14 (study #1 and #2), all (100%) Felpreva®-treated cats had lower mite counts compared to pre-treatment assessments. This was demonstrated by improved mite count scores which were significantly different from mite count scores seen in control cats. These results indicate that the product’s onset of efficacy is fast and that ear mites are rapidly killed inside the ear canal, starting within the first two weeks after treatment. Debris and cerumen scores were also improved on Day 14, though less frequently, as it was observed in only 62.5% (study #1) to 87.5% (study #2) of all Felpreva®-treated cats. This was not unexpected, as it is known that the disappearance of otoacariosis signs may take longer, and that some degree of clinical signs may still be present in cats for several days after testing negative for ear mites (Curtis, 2004).

Both studies with non-interference design (study #2 and #3) confirmed that tigolaner is the miticidal component in the combination product, shown by comparable efficacy results in Felpreva®-treated cats (efficacy of 99.6–100%) and tigolaner-treated cats (efficacy of 99.9–100%) in contrast to Profender®-treated cats (efficacy of 31.6–36.0%).

Tigolaner belongs to the class of bispyrazoles, but its systemic insecticidal and acaricidal activity is similar to that of the isoxazolines. There are three topical isoxazoline products currently marketed for cats, all of them with demonstrated activity against *O. cynotis* mites. Treatment with esafoxolaner, sarolaner or fluralaner provided efficacy rates of 97.2–99.9% for esafoxolaner (in combination with eprinomectin and praziquantel, Nexgard® Combo spot-on for cats, Boehringer-Ingelheim Animal Health; Tielemans et al., 2021), 99.2–99.6% for sarolaner (in combination with selamectin, Stronghold® Plus for cats, Zoetis; Becskei et al., 2017), and 100% for fluralaner (alone or in combination with moxidectin, Bravecto® spot-on for cats, Bravecto® Plus spot-on for cats, MSD Animal Health; Taenzler et al., 2017, Taenzler et al., 2018), when assessed between Day 28 and Day 30 after treatment. In the Felpreva® studies presented here, efficacy against ear mites was as high as 99.6–100%, suggesting that application of Felpreva® is an equally effective treatment that not only eliminates ear mite infestations from the ear canal but essentially also interrupts the life-cycle of *O. cynotis* mites in almost all cats after only one single application. Ear mites are highly contagious and easily spread in multi-cat and multi-pet environments. Therefore, prevention of disease recurrence will strongly depend on the miticidal activity of a product. Treatment with Felpreva® resulted in high miticidal efficacy without any supportive measures, such as regular cleaning of the ears, as it is often recommended for other miticidal products, especially in-ear products.

Felpreva® is presented as a spot-on solution which is an easy-to-use medicine for a stress-free management of cats. The product was safe and very well tolerated in all three studies. In combination with high ear mite efficacy, these factors are known to have a positive influence on treatment compliance and are generally considered important product characteristics for small animal veterinarians and cat owners.

## 5 Conclusions

A single spot-on administration of Felpreva® was highly effective in clearing the *O. cynotis* infestations in cats four weeks after treatment. The topical application of Felpreva® was well tolerated in cats.

## Funding

The study was funded by 10.13039/100014851Bayer Animal Health GmbH as part of the required studies for registration for Felpreva® for marketing authorisation in Europe. The funders had no role in study design, data collection and analysis, decision to publish, or preparation of the manuscript.

## Ethical approval

The studies were designed in accordance with the standards of Good Clinical Practice (VICH Guideline 9). Cats were handled in compliance with the relevant Animal Care and Use/Ethics Committee approvals. Cats were housed individually after Day 0, according to accepted local animal welfare regulations (South African National Standard SANS 10386 for study #1 and #2; US Department of Agriculture (USDA) Animal Welfare Regulations for study #3), and Ethics Committee approvals, where applicable.

## CRediT authorship contribution statement

**Katrin Blazejak:** Conceptualization, Funding acquisition, Writing – review & editing. **Alta Viljoen:** Investigation, Methodology, Resources, Data curation. **Reinier Zwiegers:** Formal analysis. **Roland Klopper:** Formal analysis. **Hannah Ringeisen:** Investigation, Methodology, Resources, Supervision. **Gabriele Petry:** Investigation, Methodology, Resources, Supervision. **David R. Young:** Investigation, Methodology, Resources. **Douglas Shane:** Investigation, Methodology, Resources. **Jennifer Spruill:** Investigation, Methodology, Resources, Supervision. **Ronald K. Tessman:** Methodology, Formal analysis, Data curation. **Terry Settje:** Methodology, Formal analysis, Data curation. **Tanja N. Knoppe:** Formal analysis, Writing – original draft, Writing – review & editing. **Norbert Mencke:** Writing – review & editing.

## Declaration of competing interests

The authors declare the following financial interests/personal relationships which may be considered as potential competing interests: Hannah Ringeisen and Gabriele Petry were employees of Bayer Animal Health GmbH, an Elanco Animal Health Company at the time while the studies reported here were conducted. Katrin Blazejak and Norbert Mencke are employees of Vetoquinol S.A., Paris, France. Tanya N. Knoppe is owner of Vet Advice, Hamburg, Germany. Alta Viljoen is an employee of Clinvet International (Pty) Ltd, Blomfontein, South Africa. Reinier Zwiegers and Roland Klopper are employees of ClinData Blomfontein, South Africa. Jennifer Spruill, Ronald K. Tessman and Terry Settje are employees of Elanco Animal Health, Greenfield, USA. David R. Young was owner of Young Veterinary Research Services, Turlock, CA, USA. Douglas Shane is employee of Young Veterinary Research Services, Turlock, CA, USA.

## Data Availability

The data supporting the conclusions of this article are included within the article. Raw data generated in the study are confidential.

## References

[bib1] Becskei C., Reinemeyer C., King V.L., Lin D., Myers M.R., Vatta A.F. (2017). Efficacy of a new spot-on formulation of selamectin plus sarolaner in the treatment of *Otodectes cynotis* in cats. Vet. Parasitol..

[bib2] Beugnet F., Bourdeau P., Chalvet-Monfray K., Cozma V., Farkas R., Guillot J. (2014). Parasites of domestic owned cats in Europe: Co-infestations and risk factors. Parasites Vectors.

[bib3] Bowman D.D., Hendrix C.M., Lindsay D.S., Barr S.C. (2002).

[bib4] Brame B., Cain C. (2021). Chronic otitis in cats: Clinical management of primary, predisposing and perpetuating factors. J. Feline Med. Surg..

[bib5] CAPC, 2019. CAPC guidelines. Otodectic mange. Companion Animal Parasite Council. https://capcvet.org/guidelines/otodectic-mite/. (Accessed 8 November 2022).

[bib6] Curtis C.F. (2004). Current trends in the treatment of *Sarcoptes*, *Cheyletiella* and *Otodectes* mite infestations in dogs and cats. Vet. Dermatol..

[bib7] Cvejić D., Hellmann K., Petry G., Ringeisen H., Hamburg H., Farkas R. (2022). Multicenter randomized, and blinded European field study evaluating the efficacy and safety of Felpreva®, a novel spot-on formulation containing emodepside, praziquantel and tigolaner, in treating cats naturally infested with fleas and/or ticks. Curr. Res. Parasitol. Vector-Borne Dis..

[bib8] Cvejić D., Mencke N., Petry G., Ringeisen H., Hamburg H., Hellmann K. (2022). Multicenter randomized, and blinded European field study evaluating the efficacy and safety of Felpreva®, a novel spot-on formulation containing tigolaner, emodepside and praziquantel, in treating cats with mixed infection with intestinal nematodes, cestodes and/or lungworms. Curr. Res. Parasitol. Vector-Borne Dis..

[bib9] Duarte A., Castro I., Pereira da Fonseca I.M., Almeida V., Madeira de Carvalho L.M., Meireles J. (2010). Survey of infectious and parasitic diseases in stray cats at the Lisbon Metropolitan Area, Portugal. J. Feline Med. Surg..

[bib10] EMA, 1994. Demonstration of efficacy of ectoparasiticides. Guideline to Directive 81/852/EEC as amended. European Medicines Agency. https://www.ema.europa.eu/en/documents/scientific-guideline/demonstration-efficacy-ectoparasiticides_en.pdf. (Accessed 8 November 2022).

[bib11] EMA, 2000. VICH GL9 Guideline on Good Clinical Practices CVMP/VICH/595/98-FINAL https://www.ema.europa.eu/en/documents/scientific-guideline/vich-gl9-good-clinical-practices-step-7_en.pdf. (Accessed 8 November 2022).

[bib12] EMA (2016). EMEA/CVMP/EWP/005/2000-Rev.3: Guideline for the testing and evaluation of the efficacy of antiparasitic substances for the treatment and prevention of tick and flea infestation in dogs and cats. https://www.ema.europa.eu/en/documents/scientific-guideline/guideline-testing-evaluation-efficacy-antiparasitic-substances-treatment-prevention-tick-flea_en-0.pdf.

[bib13] EMA (2021). EMA/532968/2021. CVMP assessment report for Felpreva® (EMEA/V/C/005464/0000). https://www.ema.europa.eu/en/documents/assessment-report/felpreva-epar-public-assessment-report_en.pdf.

[bib14] ESCCAP, 2022. Control of ectoparasites in dogs and cats. European Scientific Counsel Companion Animal Parasites ESCCAP Guideline 03, 7th ed., January 2022 https://www.esccap.org/uploads/docs/4ce0ad9k_0720_ESCCAP_GL3__English_v17_1p.pdf. (Accessed 8 November 2022).

[bib15] Fanelli A., Doménech G., Alonso F., Martínez-Carrasco F., Tizzani P., Martínez-Carrasco C. (2020). *Otodectes cynotis* in urban and peri-urban semi-arid areas: A widespread parasite in the cat population. J. Parasit. Dis..

[bib16] Genchi M., Vismarra A., Zanet S., Morelli S., Galuppi R., Cringoli G. (2021). Prevalence and risk factors associated with cat parasites in Italy: A multicenter study. Parasites Vectors.

[bib17] Jacobson L.S. (2002). Diagnosis and medical treatment of otitis externa in the dog and cat. J. S. Afr. Vet. Assoc..

[bib18] Lefkaditis M.A., Koukeri S.E., Mihalca A.D. (2009). Prevalence and intensity of *Otodectes cynotis* in kittens from Thessaloniki area, Greece. Vet. Parasitol..

[bib19] Lohse J., Rinder H., Gothe R., Zahler M. (2002). Validity of species status of the parasitic mite *Otodectes cynotis*. Med. Vet. Entomol..

[bib20] Mencke N., Blazejak K., Petry G., Hamburg H., Ringeisen H., Remer C. (2023). Immediate and long-term efficacy of Felpreva®, a new spot-on formulation containing tigolaner, emodepside and praziquantel applied as a single application to cats artificially infested with the cat flea *Ctenocephalides felis*. Curr. Res. Parasitol. Vector-Borne Dis..

[bib21] Muller G., Kirk R.R., Miller W.H., Griffin C.E., Campbell K.L. (2012). Muller and Kirkʼs Small Animal Dermatology.

[bib22] Noli C., Noli C., Colombo S. (2020). Feline Dermatology.

[bib23] Otranto D., Milillo P., Mesto P., De Caprariis D., Perrucci S., Capelli G. (2004). *Otodectes cynotis* (Acari: Psoroptidae): Examination of survival off-the-host under natural and laboratory conditions. Exp. Appl. Acarol..

[bib24] Sotiraki S.T., Koutinas A.F., Leontides L.S., Adamama-Moraitou K.K., Himonas C.A. (2001). Factors affecting the frequency of ear canal and face infestation by *Otodectes cynotis* in the cat. Vet. Parasitol..

[bib25] Taenzler J., de Vos C., Roepke R.K., Frénais R., Heckeroth A.R. (2017). Efficacy of fluralaner against *Otodectes cynotis* infestations in dogs and cats. Parasites Vectors.

[bib26] Taenzler J., de Vos C., Roepke R.K.A., Heckeroth A.R. (2018). Efficacy of fluralaner plus moxidectin (Bravecto® Plus spot-on solution for cats) against *Otodectes cynotis* infestations in cats. Parasites Vectors.

[bib27] Tielemans E., Prullage J., Tomoko O., Liebenberg J., Capári B., Sotiraki S. (2021). Efficacy of a novel topical combination of esafoxolaner, eprinomectin and praziquantel against ear mite (*Otodectes cynotis*) infestations in cats. Parasite.

[bib28] Traversa D., Morelli S., Di Cesare A., Strube C., Raue K., Bisterfeld K. (2022). Efficacy of two topical combinations containing emodepside plus praziquantel, and emodepside plus praziquantel plus tigolaner, for the treatment of troglostrongylosis in experimentally infected cats. Curr. Res. Parasitol. Vector-Borne Dis..

[bib29] Wall R., Shearer D. (2001).

[bib30] Yang C., Huang H.P. (2016). Evidence-based veterinary dermatology: A review of published studies of treatments for *Otodectes cynotis* (ear mite) infestation in cats. Vet. Dermatol..

